# Cytokines in Pain: Harnessing Endogenous Anti-Inflammatory Signaling for Improved Pain Management

**DOI:** 10.3389/fimmu.2019.03009

**Published:** 2019-12-23

**Authors:** Arden G. Vanderwall, Erin D. Milligan

**Affiliations:** ^1^Department of Neurosciences, University of New Mexico School of Medicine, Albuquerque, NM, United States; ^2^Department of Anesthesiology and Critical Care, University of New Mexico School of Medicine, Albuquerque, NM, United States

**Keywords:** cytokines, chemokines, gene therapy, chronic pain, IL-10, IL-4, TNF-α, CCL2

## Abstract

Current pain therapeutics offer inadequate relief to patients with chronic pain. A growing literature supports that pro-inflammatory cytokine signaling between immune, glial, and neural cells is integral to the development of pathological pain. Modulation of these communications may hold the key to improved pain management. In this review we first offer an overview of the relationships between pro-inflammatory cytokine and chemokine signaling and pathological pain, with a focus on the actions of cytokines and chemokines in communication between glia (astrocytes and microglia), immune cells (macrophages and T cells), and neurons. These interactions will be discussed in relation to both peripheral and central nervous system locations. Several novel non-neuronal drug targets for controlling pain are emerging as highly promising, including non-viral IL-10 gene therapy, which offer the potential for substantial pain relief through localized modulation of targeted cytokine pathways. Preclinical investigation of the mechanisms underlying the success of IL-10 gene therapy revealed the unexpected discovery of the powerful anti-nociceptive anti-inflammatory properties of D-mannose, an adjuvant in the non-viral gene therapeutic formulation. This review will include gene therapeutic approaches showing the most promise in controlling pro-inflammatory signaling via increased expression of anti-inflammatory cytokines like interleukin-10 (IL-10) or IL-4, or by directly limiting the bioavailability of specific pro-inflammatory cytokines, as with tumor necrosis factor (TNF) by the TNF soluble receptor (TNFSR). Approaches that increase endogenous anti-inflammatory signaling may offer additional opportunities for pain therapeutic development in patients not candidates for gene therapy. Promising novel avenues discussed here include the disruption of lymphocyte function-associated antigen (LFA-1) activity, antagonism at the cannabinoid 2 receptor (CB2R), and toll-like receptor 4 (TLR4) antagonism. Given the partial efficacy of current drugs, new strategies to manipulate neuroimmune and cytokine interactions hold considerable promise.

## Introduction

Pain is the evolutionarily protective perception of a nociceptive stimulus indicative of actual or potential bodily harm. However, when pain occurs outside the window of usefulness it is termed pathological. It is considered chronic when it lasts 3 months or greater (1). Chronic pain is a global problem affecting more than 1.5 billion people, and in 2015 was the leading cause of disability in most countries (2, 3). Unfortunately, clinically available medications offer insufficient long-term analgesia in many pain patients (4). Furthermore, as is the case with opioids like morphine, they can be associated with unwanted effects (e.g., sedation and constipation), rapid onset of tolerance, a high potential for abuse, and even risk of death (5).

Physiologic pain is mediated by neuron-to-neuron signaling with classical neurotransmitters and neuropeptides like glutamate, calcitonin-gene related protein (CGRP), and substance P (6). However, considerable evidence indicates that neurons are not alone in the development of pathological pain. Rather, numerous immunological pathways mediated by glia (e.g., astrocytes and microglia), immune cells, and pro-inflammatory cytokines and chemokines modify neuronal communication leading to pathological pain (7–9). Current pain therapeutics neglect the actions of these non-neuronal contributors. Therefore, development of interventions designed to target neuroimmune communication will likely improve patient outcomes.

The goal of this review is to provide an overview of the immune contributions to pathological pain with a particular focus on the role of cytokines and chemokines. The authors discuss advances in gene therapeutics and novel small molecule therapeutics that relieve chronic pain through modification of neuroimmune cytokine signaling pathways.

## Overview of Immune Contributions to Pathological Pain

### Peripheral Immune Actions in Pathological Pain

Following peripheral nerve injury or infection, receptors referred to as pattern recognition receptors expressed on immune cells are activated. Specifically, pattern recognition receptors are triggered by pathogen associated molecular patterns (PAMPs), exogenous signals indicative of pathogen invasion, or by danger associated molecular patterns (DAMPs), endogenous signals indicative of sterile tissue injury. Common pattern recognition receptors include toll-like receptors (TLRs) and Nod-like receptors (NLRs) (10). Ligand interaction with TLRs and NLRs activate pro-inflammatory intracellular pathways mediated by transcription factors like nuclear factor-κB (NF-κB) and activator protein-1 (AP-1), as well as activation of the inflammasome. This leads to increased synthesis of pro-inflammatory cytokines, including tumor necrosis factor-α (TNF-α), interleukin (IL)-6, and IL-1β. Additionally, chemotactic cytokines, referred to as chemokines, like C-C motif chemokine ligand 2 (CCL2; aka MCP-1) and C-X3-C Motif Chemokine Ligand 1 (CX3CL1; aka Fractalkine), and other immune mediators like nitric oxide (NO) are also produced (9).

Within 3 days of injury, macrophages and neutrophils are recruited to the site and secrete cytokines which aid helper T cells and B cells as well as sensitize peripheral nociceptors (11). These inflammatory mediators include pro-inflammatory cytokines like TNF-α, IL-1β, IL-6, and IL-17, nerve growth factor (NGF), serotonin, histamine, and prostaglandins like prostaglandin E2 (PGE2) (12). Activated immune cells also release anti-inflammatory molecules, such as the cytokine IL-10 and pro-resolution mediators derived from omega-3 unsaturated fatty acids (e.g., resolvins, protectins, and maresins) as a method of autoregulation in the healing process and reestablishment of homeostasis (12–14).

Interestingly, sensory neurons express receptors for many of these mediators, such as cytokine receptors (IL-1βR, TNF-αR, IL-6R, and IL-17RA), NGF receptors (TrkA), and G protein coupled receptors for serotonin, histamine, and PGE2 (12, 15). Recent reports of single cell RNA-sequencing using peripheral nociceptors located in dorsal root ganglia (DRG) have identified numerous potential neuronally expressed cytokine and chemokine receptor targets, further supporting that direct immune modulation of neuronal action is a likely driver of pathological pain (16). Activation of these receptors expressed by nearby nociceptive neurons (e.g., C fibers and Aδ fibers) leads to enhanced membrane excitability and sensitization to subsequent stimulation (15, 17). This process is termed peripheral sensitization and results in primary nociceptors that become overly responsive to previously subthreshold levels of peripheral stimulus (6).

Peripheral nerve sensitization can in turn lead to enhanced nociceptor release of excitatory neurotransmitters at their terminal synapses within the spinal cord dorsal horn, a site of significant sensory signal modulation. This enhanced signaling within the dorsal horn further sensitizes second order sensory neurons through a mechanism known as central sensitization, a neurobiological plasticity process first characterized by Woolf in the early 1980s (18). Following development of central sensitization, nociceptive neurons now potently signal to higher order structures in the brain and brainstem, resulting in pathological pain perception including hyperalgesia (enhanced response to normally noxious stimuli) and allodynia (pain in response to normally non-noxious stimuli, such as to light touch) (19). Furthermore, expansion of receptive fields is often observed, resulting in pain perception from stimulation of uninjured tissue (secondary hyperalgesia) (6, 19). Pathological pain can arise if these nociceptive pathways remain excitable beyond the period of healing.

### Central Immune Actions in Pathological Pain

Astrocytes arise from neuroepithelium and are found throughout the brain and spinal cord (20), outnumbering neurons and accounting for 40–50% of all central nervous system (CNS) glia (21). In contrast, microglia have mesodermal hematopoietic origin, constituting only 5–10% of all glia, and are considered the resident mononuclear phagocytes of the CNS (7, 20).

As with injury or infection of the CNS, damage to peripheral nerves incites glia in the spinal cord dorsal horn to express increased markers of activation (22). These spinal glia assume “activated” phenotypes, with changes in morphology (e.g., switch from highly ramified to amoeboid appearance) as well as upregulation of astrocytic activation markers like glial fibrillary acidic protein (GFAP), and microglial activation markers like complement receptor 3 (CR3), cluster of differentiation 11b (CD11b), or ionized calcium binding adaptor molecule 1 (Iba1) (8, 23, 24). Of note, the recently identified microglial-specific marker transmembrane protein 119 (TMEM119) (25) may offer newfound utility in discriminating between microglia vs. macrophages/monocytes (26–28), as prior markers are expressed by both.

#### Glial Activation in Pathological Pain

Lumbar astrocytes undergo changes in morphology and increased GFAP expression, indicating a state of increased activation following chronic constriction injury (CCI) of the rat sciatic nerve (29). Since this initial study demonstrating anatomical evidence that spinal glia play a role in pain modulation, later studies documented that spinal glia are closely associated with neural synapses, express many of the same neurotransmitter receptors as neurons, and are known to assume “activated” phenotypes in response to abnormal or heightened neuronal signaling (8, 9, 15, 23, 30–32). Astrocytes and microglia express pain-relevant neurotransmitter receptors, like AMPA (α-amino-3-hydroxy-5-methyl-4-isoxazolepropionic acid) receptors, metabotropic glutamate receptors (mGluR), purinergic receptors (e.g., P2X_4_R or P2X_7_R), or neurokinin 1 receptors (NK1R), which are activated by neurotransmitters released from presynaptic nerve terminals. These neurotransmitters include glutamate, ATP and substance P (9). Following peripheral nerve damage, neurons also release chemokines like CCL2 (33) and CX3CL1(34), and other immune mediators like colony-stimulating factor 1 (CSF-1) (35) and ATP (36, 37). These released factors potently activate astrocytes and microglia (9), which in turn release pro-inflammatory cytokines (e.g., IL-1β, TNF-α, and IL-6) (38), chemokines (e.g., CCL2) (33), ATP, excitatory amino acids (EAAs), and NO (7). As in the periphery, dorsal horn neurons express receptors for many of these factors, including the pro-inflammatory cytokines, IL-1β, TNF-α, IL-6, and IL-17 (12, 15). Activation of neuronally-expressed cytokine receptors modifies neuronal function. For example, the pro-inflammatory cytokines TNF-α and IL-1β enhance excitatory synaptic transmission and suppress inhibitory synaptic transmission in neurons of spinal cord lamina II (31, 39). Whereas, TNF-α enhances spontaneous excitatory post-synaptic current (sEPSC) frequency, IL-6 reduces frequency of the spontaneous inhibitory post-synaptic current (sIPSC). Notably, IL-1β is able to enhance both sEPSC frequency and amplitude while reducing sIPSC frequency and amplitude. Additionally, TNF-α and IL-1β enhance excitatory AMPA- and NMDA-induced currents, and IL-1β and IL-6 suppress inhibitory GABA- and glycine-induced currents (39). Therefore, activation of these cytokine receptors further sensitizes local neurons leading to a feed-forward cycle of nociceptive signaling.

### Trafficking Immune Cells and Pathological Pain

#### Macrophages in Pathological Pain

While it is clear that peripheral immune cells migrate to local sites of nerve damage, a growing body of evidence supports that they also traffic to more remote pain-relevant neural tissues and play a role in the development of pathological pain. For instance, macrophages are recruited to sites remote from the initial insult, such as to the spinal cord (40, 41) and DRG (41–43), following peripheral nerve trauma in response to chemotactic factors like CCL2 (42–44) and CX3CL1 (33, 45) derived from injured neurons with their cell bodies or central projections in these distant locations. Macrophages present in the DRG are most likely important for maintenance of chronic pain. This is supported by evidence that depletion of peripheral macrophages partially reverses established paclitaxel-induced or nerve ligation-induced mechanical hyperalgesia and reduces TNF-α expression in DRG (43, 46).

#### T Cells in Pathological Pain

In addition to macrophages, T cells may also be important in the mechanisms that underlie pathological pain. For instance, activated CD4^+^ T cells, specifically Th1 and Th17 cells, migrate to associated lumbar DRG and/or the lumbar spinal cord dorsal horn in animal models of sciatic nerve transection (47–49), spared nerve injury (50, 51), partial sciatic nerve ligation (52, 53), and sciatic nerve chronic constriction injury (54).

Increased T cell trafficking in these animal models of neuropathic pain has been associated with concurrent increases in pro-inflammatory T cell-related cytokine signaling. For example, Costigan et al. identified T cell infiltration of the spinal cord dorsal horn following a spared nerve injury (SNI) in rats with concurrent increases in spinal interferon-γ (IFN-γ) expression (50). IFN-γ is the signature pro-inflammatory cytokine released by activated Th1 cells (55). In a related study, neuropathic pain resulting from spared nerve injury is significantly diminished in both Rag1 knockout (*Rag1*^−/−^; T-cell deficient) mice and IFN-γ receptor null mutant mice as compared to wildtype controls (50).

T helper cell-mediated IL-17 expression also appears to be important for pain pathology. Compared to wild-type mice, IL-17 knockout mice display significantly less mechanical hypersensitivity as well as decreased T cell accumulation at both the site of partial sciatic nerve ligation and within the relevant lumbar DRG (L3-L5) (52). Similarly, increased CD4^+^ T cells were observed in the rat spinal cord in a model of spinal nerve ligation (e.g., peripheral nerve damage), with double-positive CD4^+^/IL-17^+^ T cells located in superficial laminae of the spinal cord dorsal horn by immunofluorescence staining (51). This was accompanied by significant upregulation of IL-17, IL-1β, and IL-6 (51).

#### Sexual Dimorphism in Pathological Pain

Much of what is known about the mechanisms of pain was identified in male animal models. However, recent reports indicate that mechanisms of pain may differ between the sexes (56, 57). While microglial activation is required for preclinical models of pathological pain in males, this does not seem to be the case for females who instead appear to be more vulnerable to the actions of T cells (54, 58–61).

Recent work from Noor et al. demonstrates that males and females exhibit divergent T cell-associated actions within discrete pain-relevant anatomic regions in a rodent model of sciatic neuropathy (54). During neuropathy, females display more profound Th17 specific responses with higher levels of IL-17A than males, both at the injured nerve and in the corresponding lumbar spinal cord. When neuropathic animals were treated with a β2-integrin antagonist, BIRT377, pain-relieved females expressed dramatically reduced IL-17A levels in sciatic and lumbar spinal cord tissues as compared to their male counterparts (for further discussion, see section “Lymphocyte Function-Associated antigen-1” below). This not only supports a role for IL-17A in mechanisms of neuropathic pain, it offers evidence of a T cell differentiation bias toward a pro-inflammatory status that is significantly greater in females than males (54).

There is growing evidence that other cytokines may also be differentially regulated in males vs. females following peripheral nerve injury. For instance, recent RNA-seq analysis of lumbar DRG following rat sciatic CCI identified that the cytokine CSF-1 is 1.7 times higher in females than males (62).

## Gene Therapeutic Modulation of Cytokines for Control of Pathological Pain

### Gene Therapeutic Approaches and Challenges

Gene therapy offers the advantage of anatomically targeted long-duration expression of a desired protein, thereby limiting possible unwanted systemic effects observed in other types of pharmacologic interventions. Currently available gene therapeutic approaches aim to treat disease through modification of gene expression by cells, often via viral transduction or non-viral transfection, though newer gene editing approaches, such as through CRISPR/Cas9, may become more common (63). To date, there are three FDA approved gene therapies, including Spinraza (nusinersen; anti-sense oligonucleotide for treatment of spinal muscular atrophy type 1) (64, 65), Kymriah (tisagenlecleucel; CAR T-cell therapy for treatment of B-cell precursor acute lymphoblastic leukemia) (66), and Luxturna (voretigene neparvovec; adeno-associated virus-mediated delivery of RPE65 for treatment of RPE65-mediated inherited retinal dystrophy) (67). Various gene therapies first began to enter clinical trials in the 1990s, but these early forays were plagued by unexpected toxicities, limited clinical benefit, and even cases of patient deaths (63). Further investigations revealed obstacles to using viral vectors, including insertional mutagenesis, immune-targeted destruction of genetically modified cells, and potentially fatal immunogenicity associated with the viral vectors themselves (68–70). While more modern viral vector approaches aim to limit these toxicities, non-viral approaches have become an appealing alternative. Specifically, viral approaches rely on viral vectors to mediate cellular transfection and subsequent gene expression. In contrast, non-viral gene therapies gain cellular access through a wide variety of mechanisms ranging from simple cellular uptake of naked plasmid DNA, to more formal packaging of genetic material within carriers like liposomes, polymers, or basic proteins to facilitate transfection (69–72). These packaging modifications are often aimed at limiting immune detection, protecting the payload (e.g., plasmid DNA) from degradation by endonucleases, targeting to the therapy to cells of interest, as well as promoting cellular entry (73).

Given that sustained functional suppression of pain-relevant pro-inflammatory cytokines may ameliorate chronic pain conditions, gene therapeutic expression of targets leading to improved anti-inflammatory function has been explored. A summary of reports in which gene therapeutics encoding anti-inflammatory mediators induce pain relief observed in pre-clinical pain models and in which changes in inflammatory mediators were also evaluated is provided here (Table 1).

**Table 1:** Anti-inflammatory gene therapies for treatment of pathological pain.

**Target**	**References**	**Vector/Delivery**	**Model organism**	**Pain model**	**Inflammatory mediators**
IL-4	Hao et al. (74)	HSV/Subcutaneous	Male Sprague-Dawley Rats	L5 Spinal Nerve Ligation	**Lumbar Spinal Cord DH:** ↓ IL-1β, p-p38, PGE2 **Lumbar DRG:**↑ IL-4
TNFSR	Peng et al. (75)	HSV/Subcutaneous	Female Sprague-Dawley Rats	Spinal Cord Injury	**Lumbar Spinal Cord:** ↓ TNF-α, p-p38 **Lumbar Spinal Cord DH:** ↓ p-p38
	Hao et al. (76)	HSV/Subcutaneous	Sprague-Dawley Rats	Sciatic Nerve Ligation	**Lumbar Spinal Cord DH**: ↓ TNF-α, IL-1β, p-p38
	Huang et al. (77)	HSV/Subcutaneous	Male Sprague-Dawley Rats	Perineural HIV gp120	**Lumbar Spinal Cord DH:** ↓ TNFα, CXCL12, CXCR4, ↑ TNFSR **Lumbar DRG:** ↓ TNF, CXCL12, CXCR4, ↑ TNFSR
	Ortmann and Chattopadhyay (78)	HSV/Subcutaneous	Sprague-Dawley Rats	Diabetic Neuropathy	**Lumbar Spinal Cord DH:** ↓ TNF-α, p-p38 **Lumbar DRG:** ↓ TNF-α, p-p38, ↑ TNFSR **Sciatic Nerve:** ↓ TNF-α
IL-10	Milligan et al. (79)	Adenovirus/Intrathecal	Male Sprague-Dawley Rats	Sciatic Inflammatory Neuropathy	**CSF:** ↓ IL-1β, ↑ IL-10
	Lau et al. (80)	HSV/Subcutaneous	Male Sprague-Dawley Rats	Spinal Cord Injury	**Lumbar Spinal Cord DH:** ↓ TNF-α
	Zheng et al. (81)	HSV/Subcutaneous	Male Sprague-Dawley Rats	Perineural HIV gp120 with DDC	**Lumbar Spinal Cord DH:** ↓ TNF-α, p-p38, CXCL12, CXCR4 **Lumbar DRG:** ↓ TNF-α, p-p38, CXCL12, CXCR4
	Zheng et al. (82)	HSV/Subcutaneous	Male Sprague-Dawley Rats	Perineural HIV gp120	**Lumbar Spinal Cord DH:** ↓ TNF-α, p-p38, CXCL12, CXCR4 **Lumbar DRG:** ↓ TNF-α, p-p38, CXCL12, CXCR4
	Milligan et al. (83)	Naked Plasmid DNA/Intrathecal	Male Sprague-Dawley Rats	Sciatic CCI	**Lumbar Spinal Cord DH:** ↑ IL-10 **Cauda Equina:**↑ IL-10
	Ledeboer et al. (84)	Naked Plasmid DNA/Intrathecal	Male Sprague-Dawley Rats	Chemotherapy-induced Peripheral Neuropathy	**Lumbar DRG:** ↓ IL-1β, p-p38, TNF-α; ↑ IL-10 **Meninges:** ↑ IL-10
	Sloane et al. (85)	Naked Plasmid DNA/Intrathecal	Male Sprague-Dawley Rats	Sciatic CCI	**CSF:** ↑ IL-10
	Soderquist et al. (86)	PLGA Encapsulated Plasmid DNA/Intrathecal	Male Sprague-Dawley Rats	Sciatic CCI	**Lumbar Spinal Cord:** ↑ IL-10 **CSF:** ↑ IL-10
	Dengler et al. (87)	Naked Plasmid DNA with D-mannose Pre-treatment/Intrathecal	Male Sprague-Dawley Rats	Sciatic CCI	**Lumbar Spinal Cord:** ↓ IL-1β, ↑ IL-10 **Lumbar DRG:** ↑ IL-10
	Vanderwall et al. (28)	Naked Plasmid DNA co-injected with D-mannose/Intrathecal	Male IL-10 Knockout Mice	Sciatic CCI	**Lumbar Spinal Cord:**↓ TNFα, CCL2; no change IL-1β, TGF-β1 **Lumbar DRG:** ↓ TNF; ↑ IL-10, TGF-β1, IL-10Rα; no change IL-1β **Cauda Equina:** ↑ IL-10 **Sciatic nerve:** ↑ TNF, IL-1β, CCL2, IFN-γ, IL-6, CXCL1, TGF-β1; no change IL-12p70

*This table includes data from reports in which gene therapeutics encoded anti-inflammatory mediators that induced pain relief in pre-clinical pain models and also evaluated changes in inflammatory mediators (e.g., cytokines, chemokines, transcription factors, etc.) in pain-relevant tissues. Relative changes are indicated in the level of inflammatory mediators identified in tissues collected from pain relieved vs. control animals*.

*CXCL1 (C-X-C motif chemokine ligand 1; aka KC/GRO), CXCL12 (C-X-C motif chemokine ligand 12; pro-inflammatory chemokine), CXCR4 (C-X-C motif chemokine receptor 4), DDC (2′,3′-dideoxycytidine; nucleoside reverse transcriptase inhibitor), IL-10Rα (IL-10 Receptor α), IL-12p70 (Interleukin-12 p70; bioactive form), PLGA (Poly lactic-co-glycolic acid)*.

### Gene Therapy for Pathological Pain

#### Interleukin-4 Signaling

Interleukin-4 (IL-4) is an important regulator of immunity, with diverse roles in processes, such as T-cell proliferation, activated B-cell stimulation, activation of macrophages, chronic inflammation, and wound repair (88). IL-4 is primarily produced by macrophages, T-cells (especially Th2 cells), mast cells, eosinophils, and basophils (89). This cytokine exerts a variety of effects through actions at two receptor subtypes. The IL-4R-I receptor is expressed by T cells, and B cells, monocytes/macrophages, and fibroblasts (90), and is involved in the regulation of maturation and proliferation of Th2 cells, and in IgE synthesis by B cells (88).

IL-4 signaling has been shown to decrease the production and actions of several pain-relevant pro-inflammatory mediators. For instance, IL-4 suppresses NLR protein 3 (NLRP3) inflammasome-dependent caspase-1 activation and the subsequent IL-1β secretion by macrophages (91). NLRP3 serves as a sensor protein capable of initiating assembly of the intracellular inflammasome protein complex, resulting in caspase-1 activation and consequent conversion of pro-IL-1β to mature bioactive IL-1β (92, 93). IL-4 has also been shown to increase expression of endogenous IL-1 receptor antagonist (IL-1ra) mRNA and protein (94), thus diminishing the actions of pro-inflammatory IL-1β. Additionally, IL-4 directly inhibits the induction of nitric oxide synthase (NOS) and levels of cyclooxygenase 2 (COX-2), thereby decreasing the production of NO and PGE2, respectively (95).

In contrast to IL-10, which is predominantly characterized as an anti-inflammatory cytokine, IL-4 exerts some pro-inflammatory actions. In vascular endothelial cells, IL-4 induces expression of CCL2 and IL-6 (96). It also synergistically increases IL-1β, TNF-α, and vascular cell adhesion protein 1 (VCAM-1; a.k.a. CD106) in lipopolysaccharide-stimulated vascular endothelium (96). IL-4-induced IL-6 expression is mediated via a reactive oxygen species (ROS)-dependent mechanism in human aortic endothelial cells (96).

#### IL-4 Gene Therapy as a Pain Therapeutic

Despite some evidence that it can serves as a facilitator of pro-inflammatory cytokine expression (97–99), IL-4 is an attractive candidate for treatment of pathological pain due to its broad spectrum of anti-inflammatory actions. Additionally, IL-4 deficiency in naïve IL-4 knockout mice exhibit increased tactile allodynia (100). However, IL-4 protein replacement therapy is precluded from use for systemic administration due to IL-4's short protein half-life as well as its pleiotropic effects on immune and vascular function (74). Therefore, gene therapeutic approaches offer the best avenue for application.

IL-4 gene therapy has been examined for control of neuropathic pain in an animal model of spinal nerve ligation. Work by Hao et al. demonstrated that herpes simplex viral vector-mediated expression of IL-4 administered after the onset of mechanical allodynia results in 5 weeks of pain relief with concurrent increases in IL-4 protein expression in DRG (74). Similar effects were observed following a repeat injection. Interestingly, prophylactic treatment at 1 week prior to sciatic nerve ligation appeared to delay the onset of mechanical allodynia by about 5 weeks (74).

IL-4 gene therapy has also been examined in an animal model of painful inflammatory arthritis (101). Intra-articular injection of IL-4 gene therapy using a retroviral vector led to a reduction in paw swelling and decreased radiographic evidence of bone destruction, though differences only achieved significance about 2 weeks post-injection and paw edema measurements were not assessed throughout the entire timecourse of the inflammatory model (101), which can last several months (102), so duration of effect is speculative. Potential sex differences in IL-4 efficacy as a therapeutic to control chronic pathological pain has not been reported.

### Tumor Necrosis Factor and Pathological Pain

Tumor necrosis factor-α (TNF-α) is a classic pro-inflammatory cytokine that exerts actions on two cell-surface receptors which are either constitutively expressed (TNFR1, p55-R) or inducible (TNFR2, p75-R), with most cells expressing constitutively low levels of TNFR1 (103). TNF-α signaling regulates apoptotic pathways, NF-κB-induced inflammation, and stress-induced activation of protein kinases (103, 104). Of note, TNF-α drives a positive regulatory feedback loop in which TNF-induced NF-κB signaling drives further expression of TNF-α (104).

Approaches that interfere with TNF-α signaling hold promise for the treatment of neuropathic pain, as demonstrated by pre-clinical work in animal models. Many studies have demonstrated that pain behaviors can be attenuated by local or spinal administration of agents that antagonize TNF-α (105–108). TNF soluble receptor (TNFSR), commercially known as etanercept, is previously approved by the FDA for treatment of painful inflammatory conditions including rheumatoid arthritis, psoriatic arthritis, and ankylosing spondylitis (109). While preclinical trials with recombinant TNFSR have shown promise, randomized clinical trials have had limited success (109). Clinical translation may be limited by the potential loss of beneficial neuroprotective and neuroregenerative properties of TNF-α, and by its impermeability at the blood-brain-barrier (9). Interestingly, while TNFR1-mediated signaling is associated with neurotoxicity, TNFR2 signaling may by neuroprotective (110–112). Some controversy over the roles of TNFR1 vs. TNFR2 in chronic pain remains (113). The role of TNF-α in pathological pain has been extensively reviewed elsewhere (104, 113).

#### TNFSR Gene Therapy as a Pain Therapeutic

Gene therapeutic approaches may bypass some of the obstacles faced by application of recombinant TNFSR. When applied subcutaneously to the hindpaw 1 week following sciatic nerve ligation, herpes simplex virus (HSV)-mediated TNFSR is associated with transient improvements in mechanical allodynia and reversal of thermal latency (76). HSV-mediated TNFSR has also been shown to improve pain behaviors in models of diabetic neuropathy, spinal cord injury, HIV gp120-mediated inflammation of the sciatic nerve, and neuropathy secondary to antiretroviral therapy (75, 77, 78).

### Interleukin-10

#### Interleukin-10 Pleiotropically Inhibits Pro-inflammatory Signaling

Interleukin-10 (IL-10) is a powerful anti-inflammatory cytokine that binds to the IL-10 receptor (IL-10R), with IL-10R1 (a.k.a. IL-10Rα) being the high affinity subunit necessary for signal transduction (114). Importantly, the IL-10R1 subunit is expressed by astrocytes, microglia, oligodendrocytes, endothelial cells, and trafficking leukocytes (115). Despite IL-10 R1 having been identified by RNA-sequencing analysis of lumbar spinal and DRG neurons in several reports (16), these findings have not yet been convincingly validated by confirmed neuronal protein expression. Two separate reports provide evidence of IL-10R1 protein expression in embryonic (E16-18) rat neurons *in vitro* (116, 117). Post-natal rodent protein analyses have been somewhat mixed, with one report identifying IL-10R1 protein expression in DRG neurons (118), and another report failing to identify IL-10R1 in brains (115). Due to ongoing uncertainty of neuronal IL-10 receptor expression, it remains possible that the pain-relieving actions of IL-10 and other anti-inflammatory cytokines may provide therapeutic benefit through actions at neuronal receptors.

IL-10 pleiotropically inhibits the actions of many pro-inflammatory factors by numerous mechanisms briefly described here, but thoroughly discussed elsewhere [for review, see (119, 120)]. IL-10 signaling leads to decreased bioavailability of pro-inflammatory cytokine proteins through upregulation of both IL-1ra and TNF-α decoy receptor (119). This is also accomplished through reduced gene transcription, decreased mRNA stability, and reduced translation for pro-inflammatory cytokines and chemokines, many of which are downstream of toll-like receptor 4 (TLR4) (121, 122). Additionally, IL-10 destabilizes mRNA transcripts for the pro-inflammatory cytokines TNF-α and IL-1β (119, 123). IL-10 signaling also induces suppressor of cytokine signaling 1 (SOCS1) and SOCS3 production, thereby further suppressing pro-inflammatory cytokine production by targeting the p65 NF-κB subunit (a key transcriptional regulator in the inflammatory process) for degradation (124).

The anti-inflammatory effects of IL-10 come about by blocking downstream signaling of TLR4, the actions of which normally promote a pro-inflammatory environment critical for the development of neuropathic pain (125). For instance, IL-10 inhibits translation of MyD88, an adaptor protein used by TLR4 that leads to pro-inflammatory cytokine production and release (126). IL-10 also increases ubiquitination and subsequent proteasomal degradation of MyD88-dependent signaling molecules (127). Interestingly, IL-10 induces microRNA-146b, which negatively regulates TLR4-mediated pro-inflammatory cytokine and chemokine induction, with targets including IL-6, TNF-α, IL-8, CCL3, CCL2, CCL7, and CXCL10 (128). IL-10 signaling also increases transcription of numerous effector proteins that inhibit TLR-induced downstream signaling molecules. For instance, IL-10 increases expression of Abin-3 and DUSP-1, leading to inhibition of p38 MAP kinase (MAPK) phosphorylation as well as increased nuclear translocation and DNA binding of p50/p50 homodimers of NF-κB, a form of NF-κB insufficient for induction of pro-inflammatory cytokine transcription (129).

#### IL-10 Gene Therapy as a Pain Therapeutic

Several studies indicate that under some chronic pain states, patients suffer from suppressed IL-10 expression (114). Similarly, animal studies also support a link between IL-10 dysregulation and pathological pain. Following peripheral nerve injury, IL-10 is briefly upregulated in what is thought to be a compensatory effort for homeostatic conditions to return. However, as pathological pain develops, IL-10 expression falls below baseline levels in pain-relevant anatomic locations (130–134). Direct application of IL-10 protein to the site of pathology is capable of producing immediate symptom relief, effects are often transient and may require repeated injections (79). These findings support that returning IL-10 expression to near-basal levels may be key to treatment for pathological pain.

Like IL-4, IL-10 has a short half-life, thus making gene therapy a desirable route of administration, particularly in patients requiring local IL-10 expression, such as in individuals with low back radiculopathy. Initial studies utilized viral vectors for lumbar spinal (intrathecal) delivery of the IL-10 gene, including non-replicating adenovirus, adeno-associated virus, and herpes simplex viral vectors. While potent pain relief was observed in animal models of chronic sciatic pain, effects were short-lived (79–82, 135, 136). The transience of the pain relief in these studies may be a result of possible complications associated with the use of a viral vector itself, such as an immune response following exposure to viral antigens (68, 120). To circumvent these possible viral obstacles, non-viral IL-10 gene therapy was explored.

Paradoxically, though non-viral gene therapies are considered the least efficient methods of gene transfer, spinal non-viral IL-10 gene therapy has been repeatedly demonstrated to provide profound and long-lasting pain relief in a variety of animal models (28, 83–87, 114, 137–139). Spinal non-viral IL-10 gene delivery in neuropathic animals produces pain relief through elevated peri-spinal IL-10 production with corresponding reduction of pro-inflammatory mediators (84, 87, 138, 140). The intriguing possibility that the protein derived from the IL-10 transgene could be capable of dimerizing with available endogenous IL-10 protein followed by receptor binding was considered a potential mechanism for the observed enduring efficacy of this unique non-viral approach. Prior work identified a non-silent point mutation in the IL-10 gene being applied in the previously published reports, and there had been some concern that it might disrupt the ability of plasmid-derived IL-10 to engage endogenous IL-10 (28). However, a recent report demonstrates that non-viral IL-10 gene therapy applied to neuropathic IL-10 knockout mice following sciatic chronic constriction injury provides long-lasting relief of allodynia, indicating that endogenous IL-10 is not required for these effects (28). Expression of the IL-10 transgene expression in pain-relieved IL-10 knockout mice was identified in lumbar DRG with concurrent increases in anti-inflammatory transforming growth factor (TGF)-β1 and decreases in pro-inflammatory TNF-α mRNA. While no IL-10 transgene was identified in the lumbar spinal cord, anti-inflammatory changes in the DRG appear to drive concordant decreases in lumbar TNF-α, CCL2, and markers of both astrocytic (GFAP) and microglial (TMEM119) activation (28). This provides compelling evidence that modification of cytokines within the DRG may be sufficient for relief of spinal mechanisms of neuropathic pain following peripheral nerve damage. To date, sex differences in IL-10 efficacy for treating chronic pathological pain have not been reported.

Non-viral IL-10 gene therapy holds significant promise for clinical translation. At this time the non-viral IL-10 gene therapy XT-150 is currently in phase I/II clinical trials (ClinicalTrials.gov Identifiers: NCT03282149; NCT03769662; NCT03477487).

### Adjuvants for Non-viral Gene Therapy

While non-viral IL-10 gene therapy has been shown to effectively relieve pathological pain in numerous animal models, initial studies required repeated large doses of DNA, thereby making clinical translation less feasible. To ameliorate the dose limitations, various carriers for non-viral gene therapy have been explored but are limited by adverse effects or added levels of complexity (71, 72, 141). Co-delivered adjuvants may offer a simpler alternative to carrier-type approaches.

#### D-mannose as an Adjuvant for Non-viral Gene Therapy

D-mannose is an inexpensive and commonly available dietary supplement (142) that has been assessed via phase III clinical trial for prevention of recurrent urinary tract infections (ClinicalTrials.gov Identifier: NCT01808755) (143). D-mannose is a known mannose receptor agonist. The mannose receptor (MR; CD206) is a c-type lectin receptor that functions in endocytosis and pathogen recognition (144). MR is expressed by macrophages, dendritic cells, and microvascular endothelial cells (145), as well as astrocytes, microglia, Schwann cells, and some neurons (146–148). MR is commonly used as a macrophage marker, though increased expression of MR is often associated with anti-inflammatory macrophages (149).

D-mannose was initially explored for use as an adjuvant due to the hypothesis that it may increase intracellular uptake of naked plasmid (87). Interestingly, D-mannose greatly increases gene therapeutic efficacy allowing for long-lasting pain relief following a single co-injection of D-mannose with plasmid DNA encoding the gene for IL-10 in neuropathic rats (87). Work by Dengler et al. also suggests that D-mannose exposure itself increases anti-inflammatory signaling, possibly through actions of endogenous IL-10. However, work by Vanderwall et al. identifies that D-mannose-mediated anti-inflammatory effects do not require IL-10, as intrathecal D-mannose alone induces transient pain relief in IL-10 knockout mice (28).

Historically MR was not considered to possess signaling ability due to the receptor's absence of a clear intracellular signaling motif. Further, if signaling was identified, it was reported as pro-inflammatory in nature. However, many of these studies were performed under inflammatory states which likely predispose MR toward pro-inflammatory signaling pathways (150–152). New evidence supports that MR activation is also capable of increasing anti-inflammatory cytokines under the right conditions (144, 153). For instance, treatment of dendritic cells with “activating” anti-MR monoclonal antibodies potently increases endogenous IL-10 and IL-1ra while decreasing IL-1β and TNF-α production (154). Furthermore, D-mannose treatment prior to LPS stimulation of RAW 264.7 mouse macrophages reduces release of pro-inflammatory TNF-α, IL-1β, and NO, but increases IL-10 secretion (87).

While D-mannose was initially explored as an adjuvant for gene therapy, its modulation of pain-relevant cytokine signaling pathways make it a novel pain therapeutic in its own right. D-mannose and MR-mediated anti-inflammatory signaling may be clinically relevant for the treatment of a variety of chronic pain conditions due to its engagement of both IL-10-independent and IL-10-mediated anti-inflammation (28, 87). These characteristics may make D-mannose useful for pain relief in patients with known IL-10 gene polymorphisms that would prevent normal IL-10 production in response to other therapeutic approaches. It may also be beneficial for treatment of pain pathologies associated with decreased IL-10 expression, like fibromyalgia (114, 155).

## Harnessing Endogenous Anti-Inflammatory Capacity for Treatment of Chronic Pain

While gene therapy offers many advantages in for treatment of chronic pain, it may not be appropriate for all patients. Therapeutics must balance between homeostasis vs. immunocompromise. For example, excessive or mistimed IL-10 secretion can inhibit a sufficient pro-inflammatory response to pathogens like *Leishmania* spp. (leishmaniasis), *Plasmodium* spp. (malaria), *T. cruzi* (Chagas disease), *Mycobacterium* spp. (tuberculosis), and lymphocytic choriomeningitis virus (lymphocytic choriomeningitis), effectively enabling the pathogens to escape immune control (156). This can result in fatal or chronic infections.

Whereas gene therapeutics are designed to be expressed independently of host transcriptional regulation, therapeutics that engage expression of endogenous anti-inflammatory functions may instead allow for dampened pro-inflammatory signaling without detriment to the body's immune-activating homeostatic mechanisms. A number of therapeutics exist that support the intriguing approach of harnessing the cell's anti-inflammatory processes. We have chosen only a subset based on recent preclinical evidence of altering glial and immune cell proinflammatory activation and corresponding regulation of anti-inflammatory cytokine production. Several promising targets for treatment of chronic neuropathic pain through engagement of endogenous anti-inflammatory capacity are reviewed below.

### Cannabinoid Receptors

The two best characterized cannabinoid receptors are CB1R and CB2R, both of which belong to the G-protein coupled receptor (GPCR) superfamily and couple to the inhibitory G_i/o_ and G_i_ proteins, respectively (157). CB1R was first discovered as the receptor for Δ9-tetrahydrocannabinol (THC), the major psychoactive ingredient in *Cannabis sativa* (158). Peripherally, CB1R is primarily expressed by neurons within the heart, small intestine, urinary bladder and vas deferens, while centrally CB1R is expressed at the highest concentrations by neurons in the cerebellum, hippocampus, basal ganglia and cerebral cortex (157, 159, 160). Clinical application of CB1R agonists are limited by development of tolerance, psychotropic effects (e.g., cognitive impairment, catalepsy, and negative impacts on learning and memory), and risk of hypothermia (157).

CB2R is abundantly expressed by peripheral immune cells (e.g., lymphocytes, neutrophils, and macrophages), and at lower levels within the CNS primarily on microglia, but has also been identified on some neurons (e.g., in the hippocampus, cortex, and substantia nigra) (159–163). CB_2_R signaling, while less well-understood, appears to primarily promote anti-inflammatory effects (164), and lack the unwanted psychotropic effects observed with CB1R activation (132). CB2R agonism has been associated with increased expression of IL-10 and decreased production of pro-inflammatory mediators like TNF-α, CCL2, and nitric oxide (164). These characteristics make CB2R a potential target for pain therapeutic development.

CB2R agonists reduce pathological pain with corresponding decreases in pro-inflammatory signaling in several animal pain models, including chronic constriction injury (132), paw incision (165), L5 spinal nerve transection (166), diabetic peripheral neuropathy (167), and chemotherapy induced neuropathic pain (168–171). The pre-clinical and clinical use of cannabinoid receptor agonists in pain has been well-reviewed elsewhere (157, 172–174).

A key CB2R ligand used in these studies, AM1710, does not cross the blood brain barrier and is 57-fold more selective for the CB2R than CB1R (175). Intrathecal AM1710 was shown to both reverse bilateral mechanical hypersensitivity in a model of peripheral nerve injury, and to prevent bilateral mechanical hypersensitivity in a model if spinal gp120 inflammatory pain. While IL-10 immunoreactivity was decreased in neuropathic animals, relief of mechanical sensitivity was associated with a return to basal levels of IL-10 expression (132). This supports that targeting the body's endogenous anti-inflammatory capabilities may relieve pathological pain through re-establishment of homeostatic conditions.

More recently, several orphan receptors, including the GPCRs GPR18, GPR55, and GPR119, have been identified as putative members of the cannabinoid receptor family, with growing evidence that GPR18 and GPR55 may be novel targets for therapeutic modulation of chronic pain (160). GPR18 is expressed in spleen, lymphocytes, neutrophils, macrophages, and lymph nodes (176–179), while GPR55 is expressed in tonsil, spleen, and by CNS neurons (160, 180, 181). The immune-relevant roles of these receptors is not well-understood, though there is some evidence to suggest that GPR55 may alleviate inflammatory pain through altered expression of IL-4, IL-10, and IFN-γ (180).

### Lymphocyte Function-Associated Antigen-1

Lymphocyte function-associated antigen-1 (LFA-1; CD11a/CD18) is a β2-integrin expressed on myeloid cells, T cells, and microglia (182–184). LFA-1 is most widely understood to be important for the leukocyte–endothelial cell interaction, which involves leukocytic rolling, firm adhesion, transmigration, and sub-endothelial migration (185). In addition to the widely characterized roles of LFA-1 in cell adhesion and migration, evidence reveals that LFA-1 expression levels following immune challenge are inversely correlated with serum IL-10 expression in experimental mice (186). Reports demonstrate LFA-1 engagement with T cells results in T cell activation, T cell differentiation, and stabilization of T cell cytokine mRNA transcripts including TNF-α (186–189). For example, LFA-1 deficient mice are resistant to LPS-induced acute liver injury, and the mechanism appears to rely on increased IL-10 expression (186). *In vitro* pretreatment of RAW 264.7 macrophages with BIRT-377, an LFA-1 antagonist, prior to LPS stimulation results in a dose-dependent increase in IL-10 protein with a simultaneous decrease in IL-1β and TNF-α (190). Activation of LFA-1 beyond T cell interaction stimulates a variety of downstream signaling cascades in a context-dependent manner modified by the local cytokine milieu (191). In point of fact, these recent reports demonstrate *in vivo* pro-inflammatory cytokine contribution driven by activated LFA-1 on innate immune cells (e.g., macrophages) and T cells during chronic disease.

The dual actions of LFA-1 in cell migration and pro-inflammatory cytokine signaling make it a reasonable target for development of pain therapeutics. The role of LFA-1 in pathological pain was first explored in work by Noor et al. that revealed prenatal alcohol exposure (PAE) predisposes adult rats to develop neuropathic pain in response to a minor injury. They found that LFA-1 expression is increased on microglia and trafficking immune cells in the lumbar spinal cords and on peripheral immune cells of neuropathic PAE rats (192). It was later identified that intrathecal or intravenous administration of BIRT-377, a small molecule non-competitive LFA-1 antagonist, relieves allodynia in neuropathic rats, which was associated with decreased immunoreactivity for spinal markers of glial activation and IL-1β and concurrent increases in IL-10 immunoreactivity (193, 194). Additionally, BIRT-377 intravenous administration in female neuropathic rats reduces Th17 cytokines and elevates FOXP3, a T cell-specific transcription factor necessary for inducing the generation of T regulatory cells characterized by their secretion of IL-10 [(193); Noor et al., under review].

Given the potential for sexual dimorphism in pathological pain, it is noteworthy that intravenous administration of BIRT-377 relieves sciatic neuropathic pain in both male and female mice to a similar degree (54). Pain relief in neuropathic mice of both sexes was concurrent with reductions in pro-inflammatory IL-1β and TNF-α and increases in anti-inflammatory IL-10 and TGF-β1. Interestingly, female-derived T cell cytokines were found to be transcriptionally regulated by LFA-1 antagonism, as demonstrated by reduced pro-inflammatory IL-17A production and increases in IL-10, TGF-β, as well as FOXP3 (54).

### The Potential Role of TLR4 Underlying Opioid-Induced Hyperalgesia

A common and unfortunate clinical side effect of chronic opioid use is the development of a paradoxical increase in pain sensitivity, termed opioid-induced hyperalgesia (195–201). Studies demonstrate in both humans and animal models that a number of key factors affect the development of opioid-induced hyperalgesia including anatomical projections from supra-spinal regions onto spinal pain projection neurons (an axonal route where pain facilitation can occur), variants in the mu-opioid receptor, sex differences, prior experience with pain, and even local activation of microglia and astrocytes (201). Given this work's emphasis on glial and immune factors, a brief review of the literature that supports opioid-induced hyperalgesia resulting from the production of pro-inflammatory immune mediators (e.g., cytokines, chemokines, and NO) that produce hyperalgesia (202) is provided below.

TLR4 actions may play a role in opioid-induced hyperalgesia. For instance, TLR4 signaling blockade attenuates opioid-induced hypersensitivity due to morphine or morphine metabolites (e.g., M3G) (203–208). Further, inhibition of glial activation (e.g., with pharmacologic glial inhibitors like minocycline and ibudilast, or by utilization of a microglial targeted Designer Receptor Exclusively Activated by a Designer Drug [DREADD] system) or direct inhibition of specific pro-inflammatory mediators (e.g., TNF-α, IL-1β, IL-6, CX_3_CL1, NOS, and p38 MAPK) after repeated morphine attenuates opioid-induced hyperalgesia (207–212).

Not only do opioids induce hyperalgesia, recent work by Grace et al., implicates that repeated opioid exposure prolongs neuropathic pain symptoms. They showed that morphine administered to neuropathic rats leads to persistent nociceptive sensitization through induction of spinal microglial NLRP3 inflammasome via activation of TLR4. This sensitization can be prevented or reversed. Naloxone is famous for its application as a rescue agent in cases of opioid overdose. While (–)-naloxone has antagonistic actions at both the μ-opioid receptor and TLR4, the (+)-naloxone isomer retains only the ability to block TLR4 signaling due to (–)-naloxone stereoselectivity at the μ-opioid receptor (213, 214). TLR4-mediated signaling has been repeatedly implicated in opioid tolerance and opioid-induced hyperalgesia (203–208, 215). These findings indicate that use of (+)-naloxone as an adjuvant to opioid therapy may enable opioid-analgesia while limiting the negative effects caused by TLR4-mediated pro-inflammatory signaling that would ordinarily result in opioid tolerance and opioid-induced hyperalgesia.

Finally, given the growing literature that supports pro-inflammatory cytokine actions in the development of opioid-induced hyperalgesia, gene therapeutics that aim to decrease such pro-inflammatory factors or increase anti-inflammatory cytokines may offer a novel tool for both the study and treatment of this unwanted side effect.

## Concluding Remarks

The worldwide opioid crisis has brought public awareness to the concept that clinically available therapeutics for pain management are not adequate and come with significant health risks. To address this, new approaches must be developed, but this cannot occur without a better understanding of the underlying mechanisms of various chronic pain pathologies. There is now a greater appreciation for actions by non-neuronal support and immune cells, including astrocytes, microglia, and leukocytes that mediate the development and maintenance of chronic pain. Here we have described what is known about the methods of immunological communication between neurons, glia, and immune cells leading to pathological pain, with a focus on the role of pro- and anti-inflammatory cytokines. We also offer potential targets for development of novel non-opioid pain therapeutics that aim to relieve pathological pain by augmenting the body's own anti-inflammatory potential. Future studies will continue to benefit from exploring the roles of neuro-immune interactions and allow for improved patient outcomes.

## Author Contributions

AV and EM contributed to all aspects of the manuscript.

### Conflict of Interest

The authors declare that the research was conducted in the absence of any commercial or financial relationships that could be construed as a potential conflict of interest.
